# Early Impact of the COVID-19 Pandemic on Congenital Heart Surgery Programs Across the World: Assessment by a Global Multi-Societal Consortium

**DOI:** 10.1177/2150135120949462

**Published:** 2020-08-26

**Authors:** Eleftherios M. Protopapas, Mauro Lo Rito, Vladimiro L. Vida, George E. Sarris, Christo I. Tchervenkov, Bohdan J. Maruszewski, Zdzislaw Tobota, Bistra Zheleva, Hao Zhang, Jeffery P. Jacobs, Joseph A. Dearani, Elizabeth H. Stephens, James S. Tweddell, Nestor F. Sandoval, Emile A. Bacha, Erle H. Austin, Kisaburo Sakamoto, Sachin Talwar, Hiromi Kurosawa, Zohair Y. Al Halees, Marcello B. Jatene, Krishna S. Iyer, Cheul Lee, Rajesh Sharma, Yasutaka Hirata, Frank Edwin, Jorge L. Cervantes, James O'Brien, James St. Louis, James K. Kirklin, The COVID-19 International Congenital Heart Surgery Taskforce

**Affiliations:** 1 Athens Heart Surgery Institute, Athens, Greece; 2 Department of Congenital Cardiac Surgery, 27288 IRCCS Policlinico San Donato, San Donato Milanese, Italy; 3 Pediatric and Congenital Cardiac Surgery Unit, Department of Cardiac, Thoracic, Vascular Sciences and Public Health, 9308 University of Padua, Padua, Italy; 4 Division of Pediatric Cardiovascular Surgery, The Montreal Children’s Hospital of the McGill University Health Centre, Montreal, Quebec, Canada; 5 Pediatric Cardiothoracic Surgery Department, Children’s Memorial Health Institute, Warsaw, Poland; 6 Children’s Heart Link, Minneapolis, MN, USA; 7 Department of Cardiothoracic Surgery, Heart Center, Shanghai Children Medical Center, National Center for Children Health, Shanghai, China; 8 Division of thoracic and Cardiovascular Surgery, Department of Surgery, University of Florida, Gainesville, FL, USA; 9 Department of Surgery, Mayo Clinic, Rochester, MN, USA; 10 University of Cincinnati, Department of Cardiac Surgery, OH, USA; 11 Congenital Heart Institute, Fundacion Cardioinfantil-Instituto de Cardiologia, Bogota, Colombia; 12 Department of Surgery, Section of Pediatric and Congenital Heart Surgery, Columbia University New York-Presbyterian/Morgan Stanley Children’s Hospital, New York, NY, USA; 13 Department of Cardiovascular Surgery, University of Louisville, KY, USA; 14 Department of Cardiovascular Surgery, Mt. Fuji Shizuoka Children’s Hospital, Shizuoka City, Japan; 15 Department of Cardiothoracic & Vascular Surgery, All India Institute of Medical Sciences, New Delhi, India; 16 Sakakibara Sapia Tower Clinic, Tokyo, Japan; 17 Heart Center, King Faisal Hospital & Research Centre, Riyadh, Saudi Arabia; 18 Heart Institute of University of Sao Paulo, Sao Paulo, Brazil; 19 Pediatric & Congenital Heart Surgery, Fortis-Escorts Heart Institute, New Delhi, India; 20 Department of Thoracic and Cardiovascular Surgery, Seoul St. Mary’s Hospital, College of Medicine, The Catholic University of Korea, Seoul, South Korea; 21 Pediatric Cardiac Surgery, Jaypee Hospital, Noida, India; 22 Department of Cardiac Surgery, University of Tokyo Hospital, Tokyo, Japan; 23 Professor & Head of Cardiothoracic Surgery National Cardiothoracic Centre, Accra, Ghana; 24 Department of Pediatric Cardiac and Congenital Heart Disease Surgery, 42705 Instituto Nacional de Cardiologia Ignacio Chavez, Mexico; 25 Division of Cardiac Surgery, 4204 Children’s Mercy Kansas City, MO, USA; 26 Division of Cardiothoracic Surgery, 9968 University of Alabama at Birmingham, AL, USA

**Keywords:** congenital heart disease (CHD), congenital heart surgery, outcomes (includes mortality, morbidity), pediatric

## Abstract

The coronavirus disease 2019 (COVID-19) pandemic currently gripping the globe is impacting the entire health care system with rapidly escalating morbidities and mortality. Although the infectious risk to the pediatric population appears low, the effects on children with congenital heart disease (CHD) remain poorly understood. The closure of congenital heart surgery programs worldwide to address the growing number of infected individuals could have an unintended impact on future health for COVID-19-negative patients with CHD. Pediatric and congenital heart surgeons, given their small numbers and close relationships, are uniquely positioned to collectively assess the impact of the pandemic on surgical practice and care of children with CHD. We present the results of an international survey sent to pediatric and congenital heart surgeons characterizing the early impact of COVID-19 on the care of patients with CHD.

## Introduction

The rapid spread of the coronavirus disease 2019 (COVID-19) pandemic has imposed major stresses on health care resources and essentially all aspects of social and economic life around the world.^1^ Although children, including those with congenital heart disease (CHD), have been relatively spared from the infectious impact of COVID, many colleagues are reporting substantial challenges in running their CHD programs. These challenges include such factors as reductions in staff, equipment, and operating room availability as these resources are reallocated to COVID-19-infected patients or the potential of such patients.^2^

The COVID-19 pandemic, caused by the novel coronavirus severe acute respiratory syndrome coronavirus-2 (SARS-CoV-2), causes mild or no symptoms in 80% of those infected.^3^ Certain populations, specifically the older age-group with significant underlying cardiopulmonary disorders, have a higher frequency of severe manifestation of the infection, including hospital admission, prolonged cardiorespiratory support, and mortality.^4,5^ Young adults (18-40 years) and children appear to have a lower incidence of severe disease. However, in a large Chinese pediatric cohort (over 2,000 children), children less than five years old, and especially infants, were more prone to severe disease than other pediatric age groups.^6–8^ Regardless of the populations affected, the pandemic has had an undeniable impact on global health care, with our respective health systems not being adequately prepared to deal with such stresses.

Over the past decade, the potential for such a global plague has become evident though never fully materialized. In 2009 and 2010, the H1N1 “swine flu” pandemic caused thousands of deaths in the United States but never approached the levels seen with COVID-19. According to the Centers for Disease Control and Prevention, from April 12, 2009, to April 10, 2010, there were “60.8 million cases (range: 43.3-89.3 million), 274,304 hospitalizations (range: 195,086-402,719), and 12,469 deaths (range: 8,868-18,306) in the United States due to the (H1N1) pdm09 virus.”^9^

Worldwide, over 14 million individuals have been infected by the novel coronavirus SARS-CoV-2 as of this writing, with 593,000 reported deaths.^10^ Given this novel viral presentation without the capacity for accurate, widespread testing in the initial phases, these numbers likely are underestimated. Most societies came to a standstill, with “stay-in-place” orders implemented across many countries. Health care systems around the world have essentially shut down normal activities, both to prepare for the care of those infected and because noninfected patients fear acquiring the disease should they enter the hospital environment. Fortunately, several countries that were affected early in this pandemic have succeeded in “flattening the curve” of the viral spread. Some are just beginning to return to a “new normal.” The lasting effects of this pandemic, both on the health of humanity and the impact on the global economy, have yet to be realized, but they will be significant.

One of the many concerns when returning to the “new normal” is the impact on patients whose operative correction or palliation was delayed during the imposed closure of congenital heart surgery programs. The possibility that patients present with the deterioration of their clinical condition may have an impact, albeit temporary, on the results in terms of morbidity and mortality and the way surgical schedules can be altered.

The objective of this effort was to assess the impact of the COVID-19 pandemic on both health care systems and individual congenital heart programs across the globe. We report the responses to the first in a series of surveys sent to congenital heart surgeons. This initial survey captured the pandemic’s direct impact at the country, institution, and program levels.

## Methods

Through a series of conference calls, a list of questions was conceived and refined by an expert panel of internationally recognized pediatric cardiologists, pediatric and congenital heart surgeons from several international organizations, and experts in global health care (Appendix A). The survey consisted of three sections. The first section captured data regarding when the virus first impacted the country and the country’s specific public health measures taken to limit the ongoing spread. The second section consisted of questions addressing hospital-specific responses to the pandemic. The final part characterized the impact of the measures imposed by hospitals on the function of the respective congenital heart surgery programs. This was the first of a series of three surveys that will be distributed over the next 12 months. Subsequent surveys will focus on the recovery of the hospital and congenital heart surgery programs and the impact this pandemic has had on patients with CHD. Several of the significant international congenital heart surgery and cardiology societies agreed to distribute the survey to its membership. The surveys were distributed via an online process and collected centrally by the European Congenital Heart Surgeons Association (ECHSA) congenital heart surgery database. Survey responses were tabulated and checked for possible duplicate submissions from the same hospital. In such duplicate responses, wherever (minor) inconsistencies were noted, the department chief’s responses were used. All surveys were used, including those with some unanswered questions. Standard descriptive statistics were used to summarize results, where appropriate. Results were presented as aggregate observations.

## Results

### Distribution of Responses

**Figure 1.**
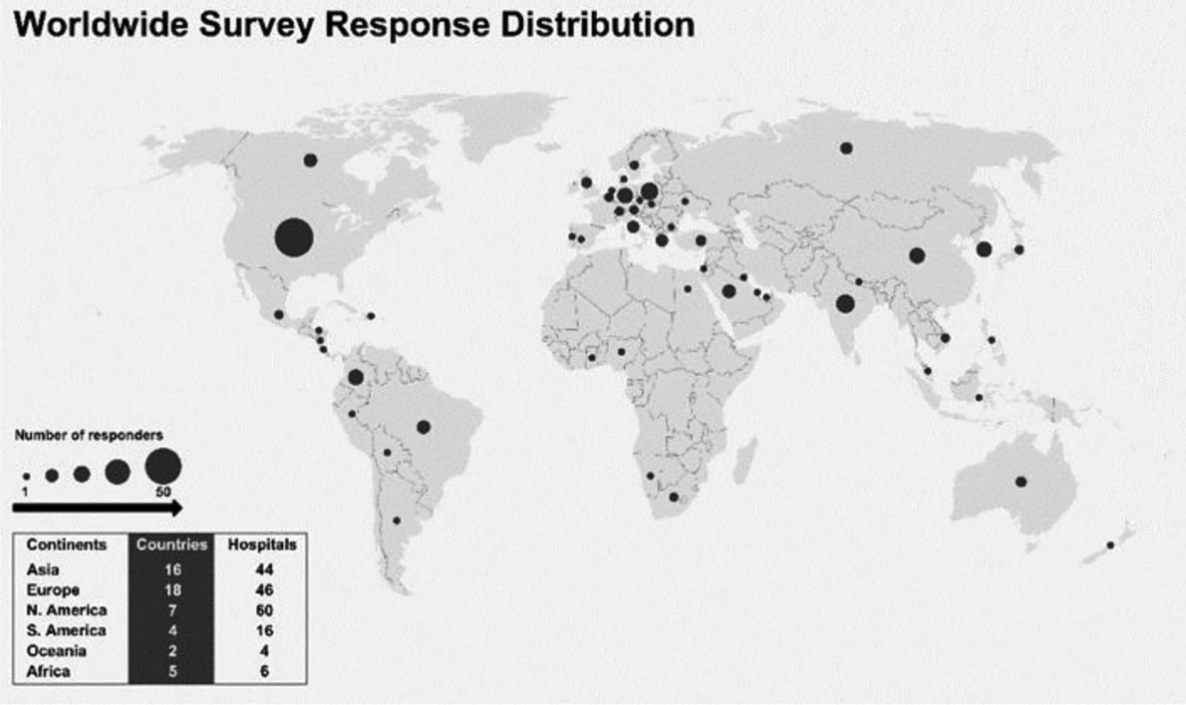
Global responses representing 52 countries, 6 continents.

A total of 202 responses from 176 hospitals in 52 countries were received during the collection period of March 27 to May 4, 2020 (Figure 1). More than one surgeon responded from the same hospital in 26 cases. The largest number of responses came from the United States, where 49 hospitals from 24 states participated. The majority of responding hospitals serve both pediatric and adult patients, while 37% (n = 65) were children’s hospitals.

### Coronavirus Disease 2019 Pandemic Impact on Countries and Hospitals

#### Social restrictions

**Figure 2.**
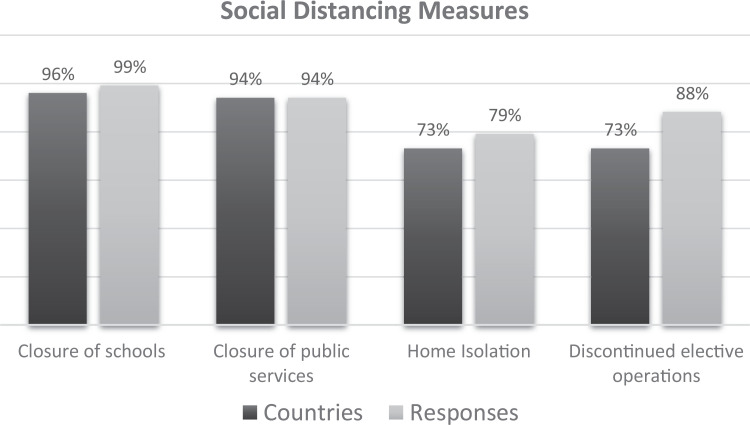
Graphical depiction of the percentage of countries and of individual hospitals in which various restrictive measures described were adopted. Differences shown for various measures between hospital and country reports are due to the fact that, in several countries, more than one center (each with possibly different adopted measures) responded.

Public health-driven societal restrictions intended to inhibit the spread of the COVID-19 infection were nearly universally imposed. Closure of schools and public services such as bars, cafés, restaurants, and sporting facilities were the most common. In only two countries, Nicaragua and Australia, schools were open when the survey response was submitted; and only in three countries (Japan, South Korea, and Sweden), public services remained open. In 73% of the countries (n = 38), citizens were ordered or advised to remain at home and the hospitals discontinued the performance of elective surgical cases. Figure 2 summarizes the restrictive measures imposed.

#### Hospital policies

**Figure 3.**
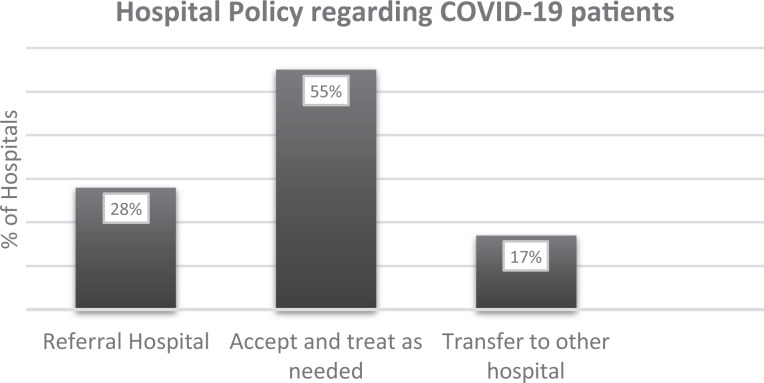
Hospital policies utilized to triage patients with COVID-19. COVID-19 indicates coronavirus disease 2019.

In response to the COVID-19 crisis, hospitals applied different policies regarding patients with COVID-19 (suspected or proven), graphically depicted in Figure 3. In a minority of hospitals (n = 31, 17.6%), the policy adopted was to transfer such patients to another hospital. Just over half of the hospitals (n = 96, 54%) accepted and treated patients who came to their emergency departments as needed. Finally, 49 (28%) hospitals were designated as a “Referral Hospital” for patients with COVID-19, meaning that its function had been adapted to preferentially treat patients with COVID-19.

#### Patient management

The large majority of hospitals (90%) dedicated spaces for patients with COVID-19 in the wards and intensive care units (ICUs). Rarely (6.3%), patients with COVID-19 were cared for in the same units as non-COVID-19 patients. Elective operations of any kind were cancelled in 91.2% (n = 161) of hospitals, and elective admissions and outpatient clinics were postponed in 85.3% (n = 150) and 78.4% (n = 138), respectively. In most cases, at the time survey responses were submitted, no shortages had been experienced in blood supply (n = 124; 71%), ICU space (n = 151; 86%), or ventilators (n = 166; 95%). Only three hospitals reported using one ventilator to support more than one patient due to ventilator shortages.

#### Extracorporeal membrane oxygenation

The availability of extracorporeal membrane oxygenation (ECMO) for patients with COVID-19 was reported in most (n = 158, 90%) of the surveyed hospitals. However, in only 31 (17.6%) of these had ECMO been used at the time of the survey, and only in adult patients suffering from COVID-19 (n = 135). None of the 158 pediatric and congenital heart surgery programs had used ECMO to support pediatric patients (at any age) suspected or diagnosed for COVID-19 infection.

### Coronavirus Disease 2019 Pandemic Impact on Congenital Heart Surgery Programs

#### Impact on surgeries

**Figure 4.**
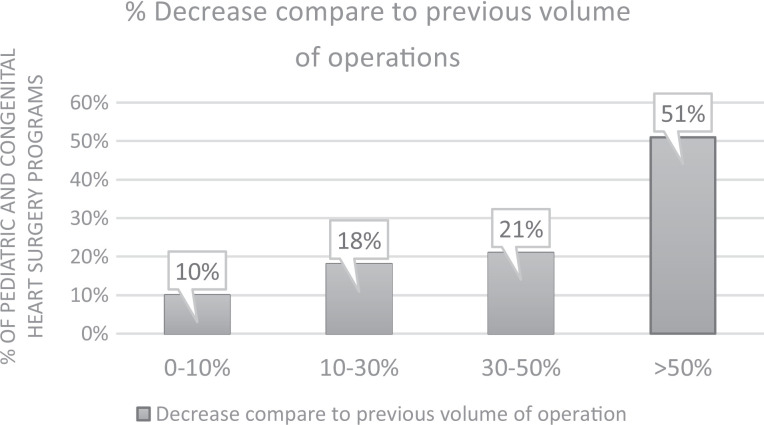
Reported decrease in congenital heart surgery program activities compared to pre-COVID-19 surgical volumes. COVID-19 indicates coronavirus disease 2019.

In almost half of the programs (n = 81, 46%), the availability of operating rooms and ICU beds was reduced due to the needs of patients with COVID-19. All elective pediatric and congenital heart operations were postponed in 152 (88%) hospitals. Only 21 (12%) institutions reported mild or no impact on surgical schedules. Half of these had been designated as referral departments for patients with CHD during the COVID-19 pandemic. The rest are in countries with different policies in restrictive measures (Sweden, Korea, and Japan), or from hospitals in countries that were either at the “beginning of the curve” (Russia, states in the United States) or “at the end,” where restrictions were lifted (Vietnam, Costa Rica). Urgent cases (cases that could not be postponed for more than one month) continued to be operated in 95% of the hospitals. Of the survey responders, the vast majority (85%) believed that postponement of elective surgeries would have a negative impact on their patients who needed cardiac operations or interventions, and they estimate that resumption of elective procedures will be delayed by one to four months (92%). The approximate reduction in the volume of operations performed in participating hospitals, as estimated by the responders, is depicted in Figure 4.

#### Impact on staff

**Figure 5.**
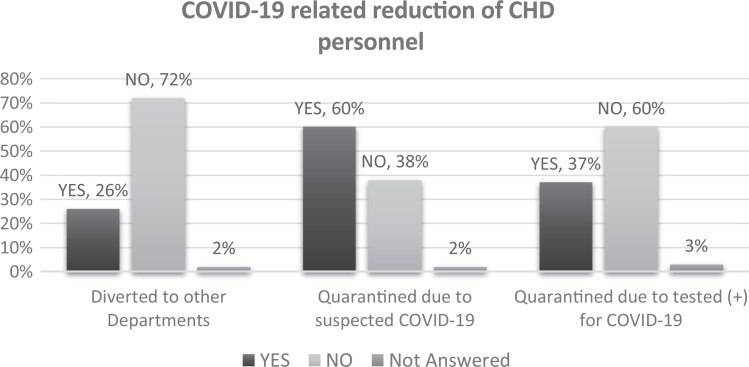
Congenital heart surgery programmatic practices to manage personnel.

In 26% of responses, doctors, nurses, and medical staff from congenital heart surgery programs were required to care for patients with COVID-19. Most congenital heart surgery programs (n = 105; 61%) reported that approximately 10% of their staff were quarantined because of suspected COVID-19 infection. In only 65 (38%) departments there were less than 10% of personnel infected. In most (68%) of the congenital programs, the COVID-19-related reductions in staff did not affect the function of their clinical services. The reduction of congenital program staff according to the cause is depicted in Figure 5.

#### Resumption of elective surgery

**Figure 6.**
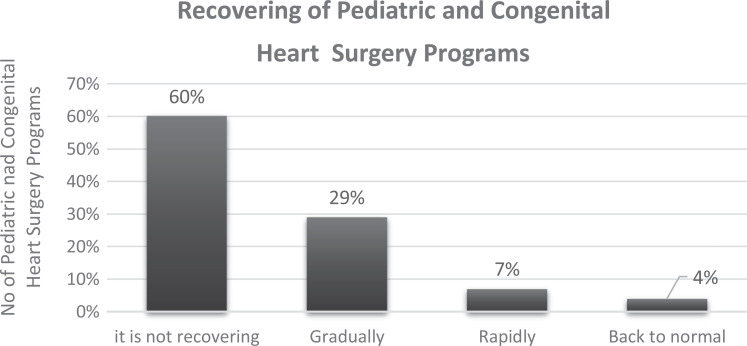
Recovery of congenital heart surgery programs as experienced by congenital heart surgeons.

**Figure 7.**
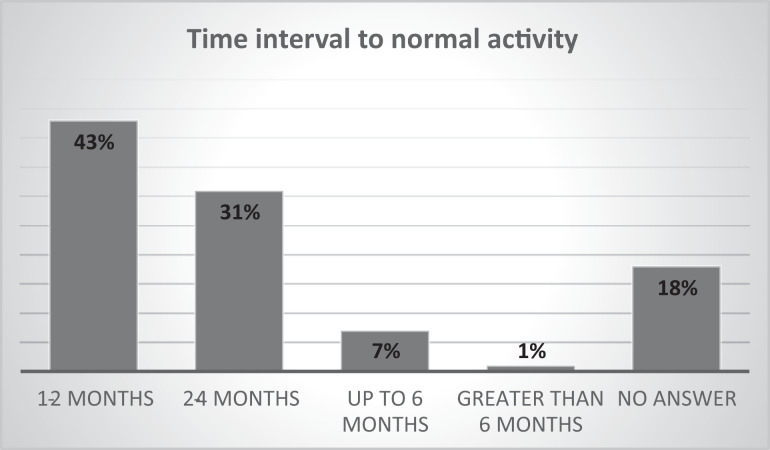
Estimated period until return of normal clinical activity with congenital heart surgery programs.

At the closure of the response period (May 4, 2020), only 20 congenital programs had resumed elective operations, mainly in Asian countries such as China, South Korea, and India. In 60% of departments, the restrictive situation remained unchanged (Figure 6). The estimated time interval required to recover normal operations postpandemic restrictions was one to two months in 43% of the programs and two to four months in 31%, as depicted in Figure 7.

#### Location of treatment for infected patients

**Figure 8.**
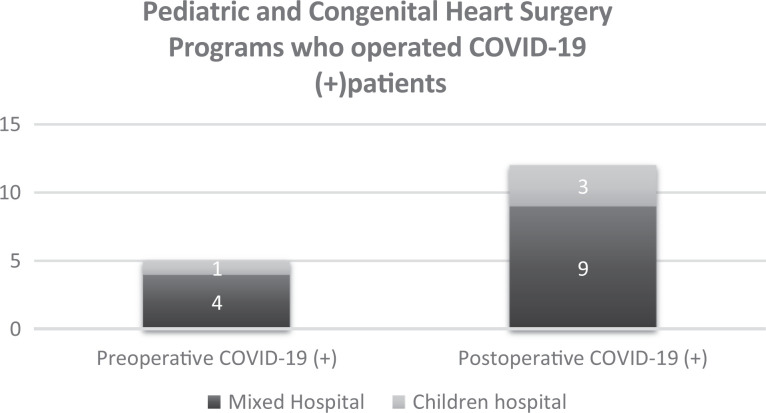
Frequency of patients with COVID-19 with congenital heart disease and type of hospital. COVID-19 indicates coronavirus disease 2019.

The cardiology departments cared for 341 patients with CHD infected with COVID-19. Of these, 38% (n = 129) were treated in home isolation and the rest hospitalized in the wards (n = 122) or the ICU (n = 90). In only five pediatric and congenital programs did COVID-19 positive patients undergo an operation (one patient in each hospital). In four of the five, the outcome was not affected. Furthermore, in 12 hospitals, COVID-19 infection was confirmed during the postoperative period in a total of 19 patients, and in one-third (n = 6) of these, the surgical outcome was adversely affected. Interestingly, the departments that operated on patients with CHD with COVID-19 infection (pre- or postoperatively confirmed) were mainly (13 out of 17) in hospitals that treat both adults and children (Figure 8).

## Comments

There is little question that the COVID-19 pandemic has had a significant impact on all aspects of our society. Most countries have shut down all public services and mandated their citizens to abide by restrictive “shelter in place” orders. The global economy has experienced a catastrophic downfall, not experienced since the Great Depression. Many health care systems have been overtaken by the severe virulence of this disease, with hospitals having either been overrun by the volumes of infected patients or implemented closure of services in anticipation of a drain on resources. There appears to be significant geographic variation as to the impact of the virus, with some regions being overwhelmed with significant mortality, while others having been barely affected. The populations affected are now better understood, with age and preinfectious medical conditions having a significant impact on morbidity and mortality. While the virus causes mild or no symptoms in 80% of those infected,^1^ specific populations (eg, elderly and patients with underlying cardiopulmonary disease) have a higher frequency of severe disease requiring hospital admission, prolonged cardiorespiratory support, and increased mortality.^2,3^ Young adults (18-40 years) and children appear to have a lower rate of severe disease. However, a large pediatric Chinese cohort (over 2,000 children) demonstrated that those less than five years old, and especially infants, were more prone to severe disease than other pediatric age groups.^4^ Although the CHD population may not be affected by this disease’s virulence, the impact created by the measures taken by countries and individual institutions to both treat those effects and to decrease the spread of the disease has not been defined.

The pediatric and congenital heart surgeons’ community is well positioned to assess the impact COVID-19 has had on congenital heart surgery and the measures enacted by their respective institutions. Compared to other medical and surgical specialties, the total numbers of active surgeons are relatively low, allowing the individual relationship to be maintained on a global scale. Organizations as the ECHSA, the World Society for Pediatric and Congenital Heart Surgery, and the Congenital Heart Surgeons Society have a long history of joint meetings, mutual bilateral exchange of knowledge and experience, and prominent cross membership. Accordingly, these societies are uniquely able to mobilize and create processes to rapidly accumulate information related to the COVID-19 pandemic and assess the institutions and congenital heart programs across the globe. These three societies, along with several other listed in Appendix B, worked through a series of video conference calls, conceived, refined, and distributed a survey with a series of questions to capture the pandemic’s impact.

Fifty-two countries were represented in the survey, illustrating the global nature of this effort. Universally, public services, including schools, were closed. Most countries (73%) enacted “stay-in-place” practices that persisted at the time of this publication. This observation was consistent with news reports across the globe. It is possible that a universal application of the “stay-at-home” order was not observed as individual countries experiencing the “peak” of the virus asynchronously. Follow-up observations will likely reveal that most countries will have instituted these policies, just at varied periods.

We observed that a majority of hospitals suspended elective operations. These measures were enacted to address either the growing numbers of infected patients requiring care or the potential reallocation of these resources. This is supported by the fact that most of the hospitals’ practice was to care for all infected patients admitted or designated as a COVID-19 referral center. Although it was anticipated that there would be a significant shortage in resources within institutions, most responses revealed minimal shortages in the hospital’s blood supply, ICU beds, and ventilators at the time of the survey. Most respondents report the ability to provide ECMO support to affected patients. At the time of response, only 17% of institutions had placed a patient with COVID-19 on ECMO, none of which were pediatric patients. As the disease process caused by this virus is becoming better understood, a late presentation in the pediatric population has been described that appears to be related to COVID-19.^6^ Multisystem inflammatory syndrome in children and adolescents temporally related to COVID-19 is a severe life-threatening multisystem inflammatory condition felt to be like Kawasaki disease and toxic shock syndrome. The impact of PMIS is not yet known but certainly has the potential to increase the need for ECMO in children with COVID-19.

Congenital heart surgery programs enacted similar restrictive measures to prepare for the anticipated loss of personnel and resources, significantly limiting elective procedures in children with CHD. All elective congenital heart surgeries were suspended in most programs across the globe. At the time of the survey, various countries were experiencing different periods on the “curve.” For example, South Korea reported a rapid increase in the number of COVID-19 positive patients, immediately enacting social restrictions. This was very early in the global acknowledgment of the existence of a pandemic. This swift action led to rapid control of the virus, with limited mortality, and expedited reopening of congenital heart surgery programs. Seemingly, the restrictions imposed on congenital heart programs may have undefined effects, including patients with CHD experiencing prolonged waiting periods. This is despite the minimal impact on programmatic resources and personnel within most programs, although a minority did report significant impact. Travel restrictions and border closures have also impacted seriously the ability of patients from countries without pediatric cardiac services to receive care either in other countries abroad, or by visiting teams.

Finally, this survey tried to answer an important question: how many of our patients (patients with CHD, followed in our departments or awaiting operation) were suffering from COVID-19 and how did infection affect their treatment? The number of operations performed on infected patients was minimal, and it seems that the outcomes were mildly affected. A more detailed registry of patients with CHD and COVID-19 will be distributed in the next period, focused on clarifying our patients’ group and delineating who is affected the most.

## Conclusion

The COVID-19 pandemic has already had a dramatic prolonged effect on our entire society. The specific impact on patients with CHD seems to be minimal, compared to the severe disease seen in older patients with underlying comorbidities. What remains unclear is the overall effect the measures enacted to control the virus, specifically, closure of most congenital heart surgery programs for nonemergency operations, will have on patients awaiting corrective or palliative cardiac operations. It would seem that these measures, particularly in children’s hospitals, were enacted in part due to a lack of knowledge of how the virus would affect this specific population. Our early data and preliminary experience with the initial wave of infection suggest that the sweeping closure of congenital heart programs may not be necessary during possible future waves of the infection. Perhaps a measured response to closures can be dictated by more objective information which will be accumulated over the next several months.

### Limitations

Studies relying on surveys to gather data are limited by their nature for several reasons. Responses are necessarily restricted to the specific answer choices listed for each question; in addition, individual respondents may interpret questions and answers differently than the survey authors intended. Distributing the survey through multiple scientific societies allows for the swift accumulation of data but does make it difficult to assess the percentage and uniformity of representation for pediatric and congenital heart surgery programs across regions, countries, or continents. This distribution method also does not preclude multiple responses being submitted from the same program. Duplicate submissions were removed from our data after answers were cross-checked for validity and only minor differences were identified. This survey did allow for some questions to be unanswered, and all survey responses were accepted regardless of the completeness of responses.

Perhaps most importantly, this survey provided a time-limited snapshot of a period in an asynchronous global event. This is unavoidable due to the nature of pandemic and the specifics surrounding the transmission of COVID-19; to alleviate this effect, we plan to distribute a series of surveys throughout the next 12 months to capture the evolution of the pandemic adequately. Finally, the ability to draw meaningful conclusions regarding the effect of COVID-19 on patients with CHD is largely precluded due to the very limited number of reports of infected patients.

## Appendix A

### COVID-19 International Congenital Heart Surgery Taskforce

Bistra Zheleva, Children’s Heart Link, Minneapolis, Minnesota; Bohdan Maruszewski, Pediatric Cardiothoracic Surgery Department Children’s Memorial Health Institute Warsaw, Poland; Cheul Lee, Department of Thoracic and Cardiovascular Surgery, Seoul St. Mary’s Hospital, College of Medicine, The Catholic University of Korea, Seoul, South Korea; Christo I. Tchervenkov, Division of Pediatric Cardiovascular Surgery, The Montreal Children’s Hospital of the McGill University Health Centre, Montreal, Quebec, Canada; Eleftherios Protopapas, Athens Heart Surgery Institute, Athens, Greece; Elizabeth H. Stephens, Department of Surgery, Mayo Clinic, Rochester, Minnesota; Emile A. Bacha, Section of Pediatric and Congenital Heart Surgery, Department of Surgery, Columbia University New York-Presbyterian/Morgan Stanley Children’s Hospital, New York, New York; Erle H. Austin, University of Louisville, Louisville, Kentucky; George Sarris, Athens Heart Surgery Institute, Athens, Greece; Hiromi Kurosawa, Sakakibara Sapia Tower Clinic, Tokyo, Japan; Hao Zhang, Department of Cardiothoracic Surgery, Heart Center, Shanghai Children Medical Center, National Center for Children Health; Katarina Hanseus, Department of Pediatric Cardiology, Skåne University Hospital, Lund, Sweden; Kisaburo Sakamoto, Department of Cardiovascular Surgery, Mt. Fuji Shizuoka Children’s Hospital, Shizuoka City, Japan; James K. Kirklin, University of Alabama at Birmingham, Birmingham, Alabama; James O’Brien, Division of Cardiac Surgery, Children’s Mercy Kansas City, Kansas City, Missouri; James St Louis, Division of Cardiac Surgery, Children’s Mercy Kansas City, Kansas City, Missouri; James S. Tweddell, University of Cincinnati, Department of Cardiac Surgery, Cincinnati, Ohio; Jeffrey P. Jacobs, Division of Thoracic and Cardiovascular Surgery, Department of Surgery, University of Florida, Gainesville, Florida; Jorge L. Cervantes, Cirugía Cardíaca Pediátrica y Adultos.Maestría en Gestión Directiva en Salud Department de Cirugía Cardíaca Pediátrica y Cardiopatías Congénitas. Instituto Nacional de Cardiología “Ignacio Chávez”, México; Joseph A. Dearani, Department of Surgery, Mayo Clinic, Rochester, Minnesota; Jose Fragata, Hospital de Santa Marta/Hospital CUF, Miraflores-Alges, Portugal; Marcelo Jatene, Heart Institute of University of Sao Paulo, Sao Paulo, Brazil; Marshall L. Jacobs, Division of Cardiac Surgery, Johns Hopkins University, Baltimore, Maryland; Mauro Lo Rito, Department of Congenital Cardiac Surgery IRCCS Policlinico San Donato, San Donato Milanese, Italy; Nestor F. Sandoval, Congenital Heart Institute Fundacion Cardioinfantil-Instituto de Cardiologia, Bogota, Colombia; Richard A. Jonas, Department of Cardiovascular Surgery, Children’s National Medical Center, Washington DC; Rajesh Sharma, Pediatric Cardiac Surgery, Jaypee Hospital, Noida, India; Yasutaka Hirata, University of Tokyo Hospital, Tokyo, Japan; Vladimiro Vida, Paediatric and Congenital Cardiac Surgery Unit Department of Cardiac, Thoracic, Vascular Sciences and Public Health University of Padua, Padua, Italy; Zdzislaw Tobota, Pediatric Cardiothoracic Surgery, Department Children’s Memorial Health Institute, Warsaw, Poland; Kirsten Finucane, Auckland City Hospital, Auckland, New Zealand; Frank Edwin, Professor & Head of Cardiothoracic Surgery National Cardiothoracic Centre, Accra, Ghana; Darshan Reddy, Ethekwini Hospital and Heart Center, Durban, South Africa; Krishna Iyer, Executive Director, Pediatric & Congenital Heart Surgery, Fortis Escorts Heart Institute, New Delhi, India; Sachin Talwar, All India Institute of Medical Sciences, New Delhi, India.

## Appendix B

### Participating Societies

#### Main sponsors

Congenital Heart Surgeons’ Society

European Congenital Heart Surgeons Association

World Society for Pediatric and Congenital Heart Surgery

#### Also participating

Association for European Pediatric and Congenital Cardiology

Japanese Society for Cardiovascular Surgery

Korean Society for Thoracic and Cardiovascular Surgery

Pediatric Cardiac Society of India

## Abbreviations and Acronyms

CHD: congenital heart disease
COVID-19: coronavirus disease 2019
ECHSA: European Congenital Heart Surgeons Association
ECMO: extracorporeal membrane oxygenation
ICU: intensive care unit
PMIS: pediatric multisystem inflammatory syndrome
SARS-CoV-2: severe acute respiratory syndrome coronavirus-2

## References

[1] Clerkin KJ Fried JA Raikhelkar J, et al. Coronavirus disease 2019 (COVID-19) and cardiovascular disease [published online ahead of print Mar 21, 2020]. Circulation. 2020;141(20):1648–1655. doi:10.1161/CIRCULATIONAHA.120.046941 32200663 10.1161/CIRCULATIONAHA.120.046941

[2] Levy E Blumenthal J Chiotos K Dearani JA COVID-19 FAQ’s in pediatric cardiac surgery [published online ahead of print Apr 21, 2020]. World J Pediatr Congenit Heart Surg. 2020;11(4):485–487. doi:10.1177/2150135120924653 32316830 10.1177/2150135120924653 PMC7444019

[3] Wu Z McGoogan JM Characteristics of and important lessons from the coronavirus disease 2019 (COVID-19) outbreak in China: summary of a report of 72314 cases from the Chinese center for disease control and prevention [published online ahead of print Feb 24, 2020] JAMA. 2020 doi:10.1001/jama.2020.2648 10.1001/jama.2020.2648 32091533

[4] Arentz M Yim E Klaff L, et al. Characteristics and outcomes of 21 critically ill patients with COVID-19 in Washington State [published online ahead of print Mar 19, 2020]. JAMA. 2020;323(16):1612–1614. doi:10.1001/jama.2020.4326 32191259 10.1001/jama.2020.4326 PMC7082763

[5] Wang D Hu B Hu C, et al. Clinical characteristics of 138 hospitalized patients with 2019 novel coronavirus-infected pneumonia in Wuhan, China [published online ahead of print Feb 7, 2020]. JAMA. 2020;323(11):1061–1069. doi:10.1001/jama.2020.1585 32031570 10.1001/jama.2020.1585 PMC7042881

[6] Dong Y Mo X Hu Y, et al. Epidemiological characteristics of 2143 pediatric patients with 2019 coronavirus disease in China. Pediatrics. 2020 doi:10.1542/peds.2020-0702

[7] Shi S Qin M Shen B, et al. Association of cardiac injury with mortality in hospitalized patients with COVID-19 in Wuhan, China [published online ahead of print Mar 25, 2020]. JAMA Cardiol. 2020;5(7):802–810. doi:10.1001/jamacardio.2020.0950. 32211816 10.1001/jamacardio.2020.0950 PMC7097841

[8] Shekerdemian L Mahmood N Wolfe K, et al. Characteristics and outcomes of children with coronavirus disease 2019 (COVID-19) infection admitted to US and Canadian pediatric intensive care units [published online ahead of print May 11, 2020]. JAMA Pediatr. 2020 doi:10.1001/jamapediatrics.2020.1948 10.1001/jamapediatrics.2020.1948 PMC7489842 32392288

[9] Verdoni L Mazza A Gervasoni A, et al. An outbreak of severe Kawasaki-like disease at the Italian epicenter of the SARS-CoV-2 epidemic: an observational cohort study [published online ahead of print May 13, 2020]. Lancet. 2020 395(10239):1771–1778. doi:10.1016/S0140-6736(20)31103-X 32410760 10.1016/S0140-6736(20)31103-X PMC7220177

[10] Centers for Disease Control and Prevention. H1N1 pandemic (H1N1pdm09 virus). 2009 Updated June 11, 2019. Accessed April 7, 2020 https://www.cdc.gov/flu/pandemic-resources/2009-h1n1-pandemic.html

[11] Lin EE Blumberg TJ Adler AC, et al. Incidence of COVID-19 in pediatric surgical patients among 3 US children’s hospitals [published online ahead of print June 04, 2020]. JAMA Surg. 2020:e202588 doi:10.1001/jamasurg.2020.2588 10.1001/jamasurg.2020.2588 PMC7273313 32496527

